# Sequence of chondrocranial development in basal anurans—Let’s make a cranium

**DOI:** 10.1186/s12983-022-00462-z

**Published:** 2022-05-03

**Authors:** Paul Lukas, Janine M. Ziermann

**Affiliations:** 1 Institute of Zoology and Evolutionary Research, Friedrich-Schiller-University, Jena, Germany; 2 Howard University College of Medicine, 520 W St NW, Washington, DC 20059 USA

**Keywords:** Viscerocranium, Chondrocranium, Neurocranium, Developmental patterning, Developmental sequence

## Abstract

**Background:**

The craniofacial skeleton is an evolutionary innovation of vertebrates. Due to its complexity and importance to protect the brain and aid in essential functions (e.g., feeding), its development requires a precisely tuned sequence of chondrification and/or ossification events. The comparison of sequential patterns of cartilage formation bears important insights into the evolution of development. *Discoglossus scovazzi* is a basal anuran species. The comparison of its chondrocranium (cartilaginous neuro- & viscerocranium) development with other basal anurans (*Xenopus laevis*, *Bombina orientalis*) will help establishing the ancestral pattern of chondrification sequences in anurans and will serve as basis for further studies to reconstruct ancestral conditions in amphibians, tetrapods, and vertebrates. Furthermore, evolutionary patterns in anurans can be studied in the light of adaptations once the ancestral sequence is established.

**Results:**

We present a comprehensive overview on the chondrocranium development of *D. scovazzi.* With clearing and staining, histology and 3D reconstructions we tracked the chondrification of 44 elements from the first mesenchymal Anlagen to the premetamorphic cartilaginous head skeleton and illustrate the sequential changes of the skull. We identified several anuran and discoglossoid traits of cartilage development. In *D. scovazzi* the mandibular, hyoid, and first branchial arch Anlagen develop first followed by stepwise addition of the branchial arches II, III, and IV. Nonetheless, there is no strict anterior to posterior chondrification pattern within the viscerocranium of *D. scovazzi*. Single hyoid arch elements chondrify after elements of the branchial arch and mandibular arch elements chondrify after elements of the branchial arch I.

**Conclusions:**

In Osteichthyes, neurocranial elements develop in anterior to posterior direction. In the anurans investigated so far, as well as in *D. scovazzi*, the posterior parts of the neurocranium extend anteriorly, while the anterior parts of the neurocranium, extend posteriorly until both parts meet and fuse. Anuran cartilaginous development differs in at least two crucial traits from other gnathostomes which further supports the urgent need for more developmental investigations among this clade to understand the evolution of cartilage development in vertebrates.

## Background

The cartilaginous cranium or chondrocranium in jaw bearing vertebrates follows a basic anatomical pattern. There are usually upper and lower jaws, nose, eye and ear capsules, and several elements surrounding the brain at least partially [1, 2]. The number of elements differs between species and the extent of fusion or ossification later in development is variable. Several elements can be correlated with the pharyngeal arch they derive from (e.g., [3]). The jaws derive from mesenchyme of the first or mandibular arch, the hyoid elements derive from the 2nd or hyoid arch, and branchial (gill bearing) or posterior pharyngeal elements derive from arches 3 to 6 (in some vertebrates more arches are present) (e.g., [4]).

Amphibians are the most basal four-legged ‘terrestrial’ vertebrates (Tetrapoda) and comprise salamanders, frogs, and caecilians. Frogs are distributed worldwide except at the poles and Greenland, and inhabit a wide range of ecosystems [5, 6]. Most frogs have a biphasic lifestyle; that is, they pass through a free-swimming aquatic larval (tadpole) stage which is connected via a remodeling process called metamorphosis to the often terrestrial adult stage (frog). Tadpoles have mostly cartilaginous cranial elements; hence their heads are mainly supported by chondrocranial elements. Therefore, the chondrocranium sets the baseline for further development of the head [7]. Bony elements do not occur until the onset of metamorphosis. The tadpoles are as diverse as the adults with tiny predatory, giant megalophagous, medium sized scraping or filter feeding tadpoles and many other varieties [6]. All the different feeding types are associated with adaptations in the cranial skeleton and musculature.

All differences in mature larvae have to be caused by differences during the development. Previously, Ziermann et al. [8] studied the sequence of development of head muscles in larvae of frogs and compared these between each other and with salamanders and the Australian lungfish. They did not find any correlation of developmental sequence and feeding style, size, or habitat. However, they found that cephalic muscles tend to develop from anterior to posterior with 1st and 2nd arch-derived muscles developing before the 3rd pharyngeal arch-derived muscles, and these before the 4th and 5th pharyngeal arch muscles. They also described that there is a pattern of the muscles developing and differentiating first externally (more superficial) before medially (deeper) located muscles. Lastly, muscles tend to develop from their origin towards their insertion [8]. Several other studies in other species confirmed the general trends (chick: [9]; lungfish: [10, 11]; small spotted cat-shark: [12]); but although pointed out that the mandibular arch seems to develop more randomly than the other arches [12], which could support the hypothesis that the mandibular arch was secondarily added to the pharyngeal or branchial arches [13].

Numerous investigations on the chondrocranial development are available and numerous species from all major vertebrate clades were added to the account recently [14–23, 23, 24]. The early chondrocranial development is similar within birds and variation relates to developmental patterns rather than to the presence or absence of specific elements [24]. A posterior anterior direction of chondrification of the skull was described in birds and squamates [19, 23, 24] whereas at least the cartilaginous visceral skeleton of several vertebrates develops in anteroposterior direction [14, 17, 18, 20]. Shared features of chondrocranial development as well as sequential differences are helpful for further analyzing phylogenetic relation and evolutionary pathways of cartilaginous development.

Although ossification sequences are available for numerous frog species, investigations on the sequence of larval cartilage development are scarce. The sequence of development for chondrocranium elements was recently studied for two basal frog species (*Xenopus laevis* and *Bombina orientalis*). These studies identified several patterns which may be candidates for the basal pattern of cartilage formation in anurans [21, 25]. (1) The first mesenchymal Anlage which appears during the chondrocranial development is the Anlage of the ceratohyal, which is also the first structure that chondrifies. (2) Anterior to posterior sequences of chondrification are present in neurocranial structures which encapsulate the sensory organs and the brain, the neurocranium-anchoring processes of the palatoquadrate, and the four ceratobranchials that form the branchial basket.

**Fig. 1:**
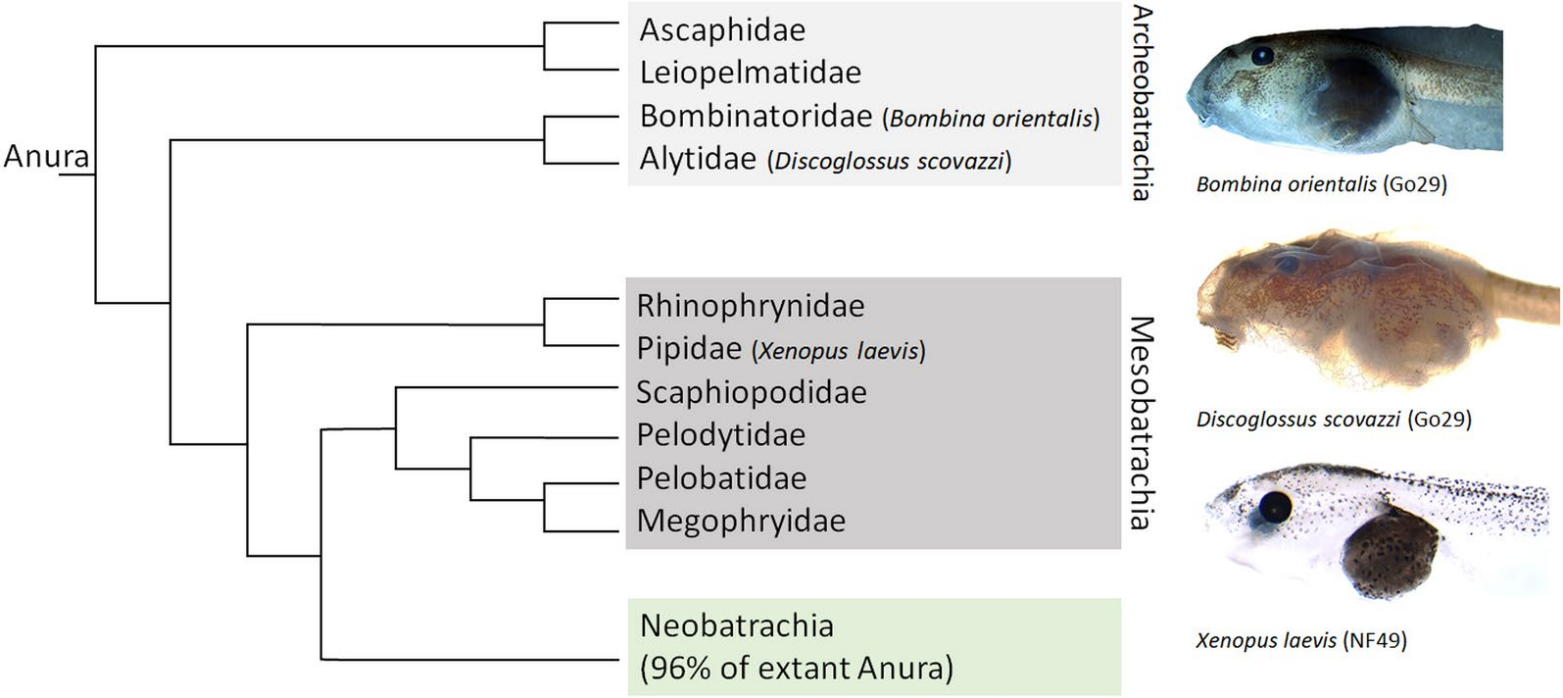
Cladogram showing the relationship of extant anuran families for the paraphyletic suborders Archeobatrachia and Mesobatrachia as well as the position of the three species studied here. The Neobatrachia are the most modern and monophyletic anuran suborder and comprise by far the most extant frog species. The relationships are based on Hime et al. [36]

In order to analyze if general developmental patterns can be identified or if there is a correlation with phylogeny, lifestyle, and/or size, we will here lay the groundwork for a larger scale comparative development study. We compare the sequence of chondrocranial development in three species *Discoglossus scovazzi*, *Xenopus laevis*, and *Bombina orientalis* (Fig. 1). *X. laevis* and *B. orientalis* were previously described [21, 25] and we only updated their developmental sequences where needed. The chondrocranium development of *D. scovazzi* will be described in detail.

The present work provides a comprehensive overview on the cartilage development of *D. scovazzi* from the first mesenchymal Anlagen until the premetamorphic cartilaginous head skeleton. This overview completes recent and sets the standards for future investigations on the chondrocranial development of larval anurans. Furthermore, we propose a defined set of structures, abbreviations and a reliable workflow for the analysis of this development. We present baseline data which is intended to be used in future comparative analyses of the cartilaginous development in anurans as well as in other tetrapods. Such extensive analyses should shed light on the evolution of the cartilaginous development during ontogenesis. In the end we want to encourage scientists to not only rely on the description of single stages when describing tadpole morphology. A sequential description of the development, no matter if muscles, cartilages, nerves or other morphological structures are investigated, bears much more information and will help to further uncover the underlying pathways of the evolution of development.

## Results

### Development of *Discoglossus scovazzi* (Table 1)

#### Infrarostral cartilage

**Fig. 2:**
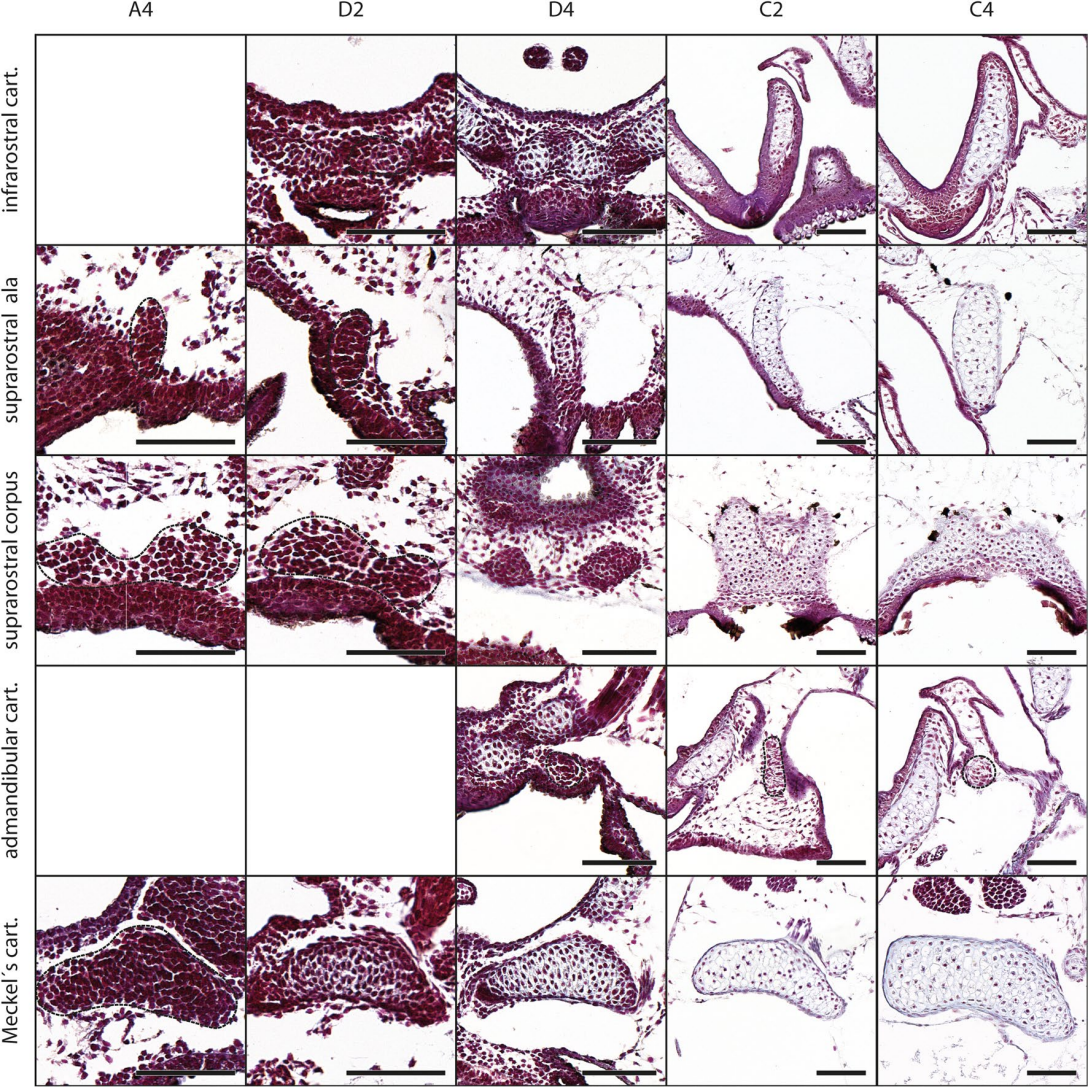
*D. scovazzi*, Cartilaginous development of mandibular arch derived structures. Transverse histological sections of Stage A4 (first column), Stage D2 (second column), Stage D4 (third column), Stage C2 (fourth column) and Stage C4 (fifth column) show the chondrification process of the infrarostral cartilage (cart.), the suprarostral ala, the suprarostral corpus, the admandibular cartilage and Meckel's cartilage. Difficult observable structures are highlighted by dashed lines. Scale bars indicate 100 μm

**Fig. 3:**
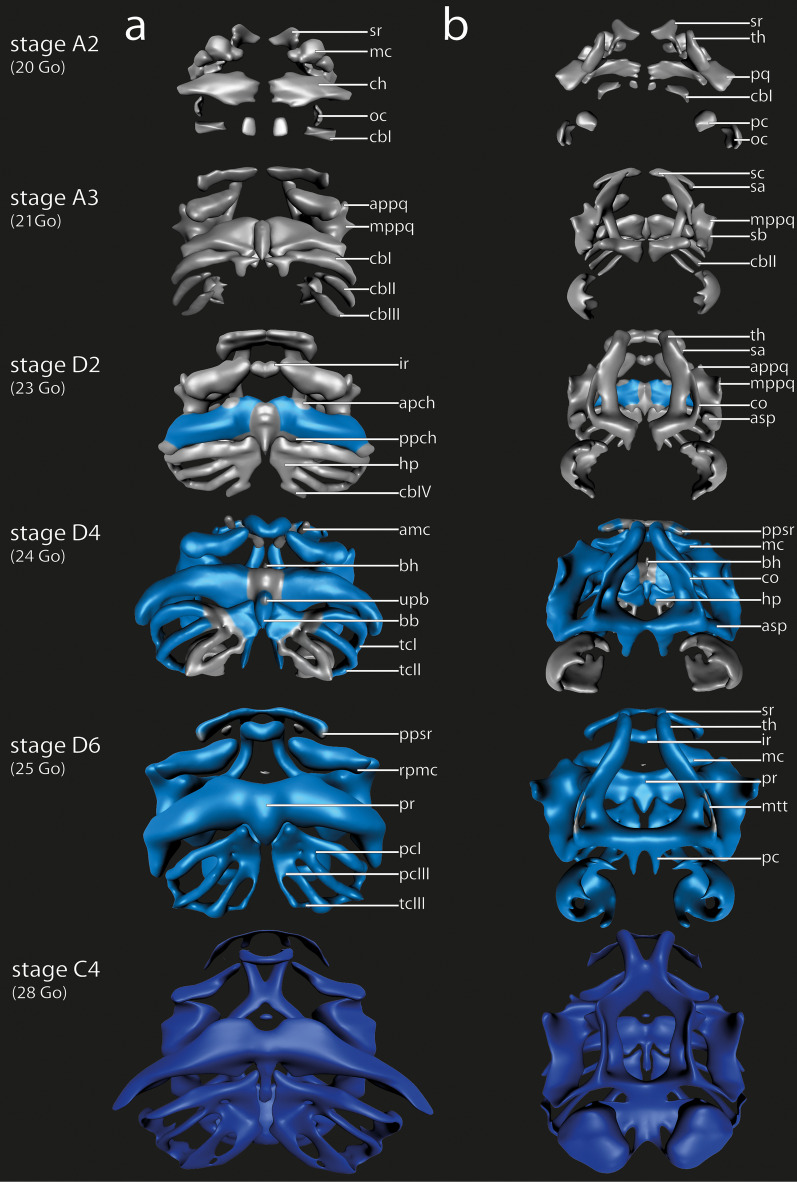
*D. scovazzi*, 3D reconstructions of the head skeleton of larvae showing the cartilaginous development during Stages A2, A3, D2, D4, D6 and C4 (top to bottom) in ventral (**a**) and dorsal (**b**) view. The 3D reconstructions are color-coded, according to the distinct stage of cartilaginous development: “Light gray” means Anlagen are visible as mesenchymal cell clusters; “light blue” means that condensed precartilaginous cell clusters containing chondroblasts are visible, “dark blue” means that the respective cartilage contains chondrocytes rich in cytoplasm and bordered by a distinct perichondrium. Sizes were adjusted. amc, admandibular cartilage; apch, anterior process of ceratohyal; appq, articular process palatoquadrate; asp, ascending process; bb, basibranchial; bh, basihyal; cbI-IV, ceratobranchial I–IV, ch, ceratohyal; co, orbital cartilage; hp, hypobranchial plate; ir, infrarostral cartilage; mc, Meckel´s cartilage; mppq, muscular process palatoquadrate; mtt, medial tectal taenia; oc, otic capsule; pc, parachordal cartilage; pcI, proximal commissure I; pcIII, proximal commissure III; ppch, posterior process ceratohyal; ppsr, posterior process suprarostral cartilage; pq, palatoquadrate; pr, pars reuniens; rpmc, retroarticular process Meckel´s cartilage; sa, suprarostral ala; sb, subocular bar; sc, suprarostral corpus; sr, suprarostral cartilage; tcI-III, terminal commissure I-III; th, trabecular horn; upb, urobranchial process

There is a single, V-shaped infrarostral cartilage in *D. scovazzi*. It is connected laterally to the paired Meckel’s cartilage via an intramandibular commissure and the intramandibular joint. The first traces of the infrarostral appear at stage D2 (Fig. 2) when the majority of cartilages are already present as mesenchymal Anlagen or differentiated chondroblasts (Fig. 3a). The mesenchymal Anlage of the infrarostral develops ventral to the anteroventral margin of the oral cavity and medially to the mesenchymal Anlagen of the Meckel´s cartilages (Fig. 2). The Anlage is already V- shaped. At stage D3 the intramandibular commissure becomes visible, which connects the dorsolateral part of the infrarostral and the ventromedial part of Meckel´s cartilage. The number of chondroblasts increase in the otherwise unchanged infrarostral so that in stage D4 only the median part still consists of dense mesenchymal cells. At the transition from stage D4 to C1 the chondroblasts differentiate into chondrocytes. In stage C2 the whole cartilage is surrounded by a distinct perichondrium. The medial parts of the infrarostral cartilage are connected by a medial synchondrosis. The small dorsolateral margin where the intramandibular joint develops chondrifies at stage C3.

**Table 1:**
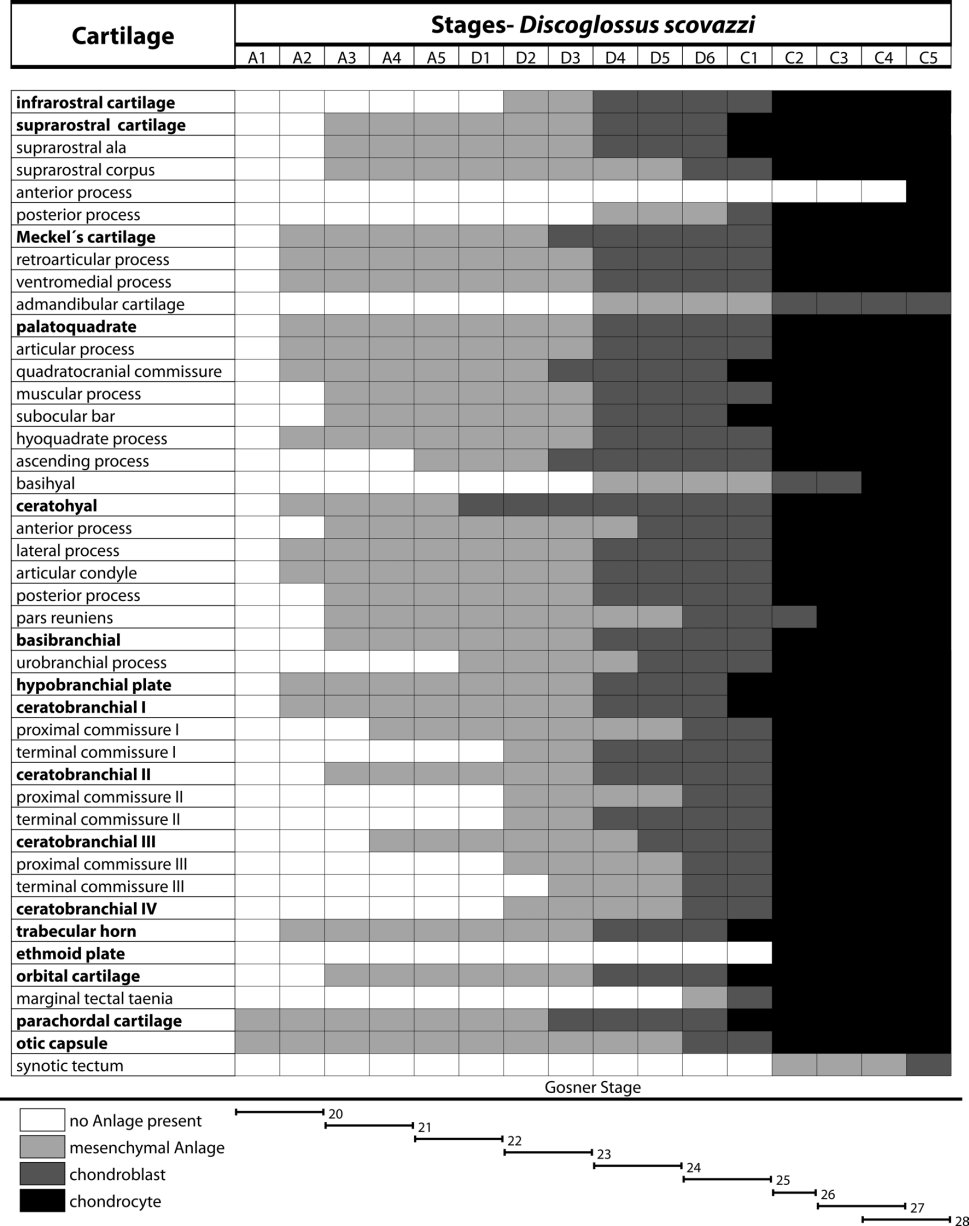
*D. scovazzi*, sequence of occurrence of cartilaginous development in tadpoles

#### Admandibular cartilage

The admandibular cartilages are small, vertically oriented rods between the suprarostral and the infrarostral cartilages. The rostrolateral tip of the admandibular cartilage is connected to the dorsomedian surface of Meckel´s cartilage through the small mandibulo-admandibular ligament. The mesenchymal Anlage is spherical and appears at stage D4 lateral to the infrarostral cartilage and ventromedial to Meckel´s cartilage (Fig. 2). The posterodorsal surface of the admandibular borders on the ventromedial surface of Meckel´s cartilage and does not interfere with the articulation between Meckel's cartilage and infrarostral cartilage. During further differentiation the admandibular Anlage extends ventrally and is rod-shaped when the first chondroblasts appear at stage C2 (Figs. 2 and 3a). The dorsal tip is in close proximity to the ventromedial process of Meckel´s cartilage. A differentiation into chondrocytes was not observed in the investigated stages.

#### Suprarostral cartilage

The plate-like suprarostral cartilage is the anteriormost cartilage. It supports the upper lip and consists of a median corpus and two lateral alae. Two dorsal projections of the corpus articulate with the anterior part of the trabecular horns. The alae emerge from the lateral margin of the corpus, bend posterolaterally and bear a short posterior process. The mesenchymal anlagen of the two alae and the corpus develop at stage A3 as two separated Anlagen which surround the anterior part of the oral cavity laterally and dorsally (Fig. 3a). Until stage A5 the mesenchymal Anlagen fuse medially and the Anlage acquires the typical plate-like shape of the suprarostral cartilage. At stage D4 the cells of the alae differentiate into chondroblasts (Fig. 2) and the mesenchymal Anlage of the posterior process arises on its posterodorsal margin. The whole Anlage of the suprarostral cartilage continues to extend laterally. This process lasts until stage D6, at which point the corpus of the suprarostral consists of chondroblasts. The alae of the suprarostral are among the first structures which consist mostly of chondrocytes at stage C1. The suprarostral corpus and the posterior processes of the alae chondrify at stage C2 (Fig. 2). This stage marks the differentiation of the majority of the cartilaginous structures of *D. scovazzi* into proper cartilages. A second, more anterior situated, posterior process emerges from the posterodorsal margin of the ala at stage C5.

#### Meckel's cartilage

Meckel's cartilage is the ventral component of the mandibular arch and forms part of the lower jaw in many vertebrates. The lower jaw of many anuran larvae consists of two elements, the medial and more anterior infrarostral cartilage and the more lateral and more posterior located paired Meckel’s cartilage. The latter articulates with the palatoquadrate via the mandibular joint. In *D. scovazzi* the Meckel’s cartilage is slightly sigmoid and typically horizontally oriented. It consists of the clearly distinguishable retroarticular process dorsolaterally and an inconspicuous ventromedial process. The mesenchymal Anlagen of Meckel's cartilage and its processes are among the earliest visible skeletal structures during skeletogenesis. They arise at stage A2 as two distinct condensations beneath the ventrolateral margin of the oral cavity (Fig. 3a). Dorsolaterally the Anlagen are bordered by the mesenchymal Anlagen of the mandibular levator muscles. Medially the contralateral Anlagen are almost in contact with each other. Both Anlagen of Meckel's cartilage widen vertically during further development and the median gap between both Anlagen enlarges. The cells of Meckel’s corpus differentiate into chondroblasts at stage D3. The processes remain as mesenchymal Anlagen until stage D4, when the ventromedial process articulates with the lateral margin of the infrarostral cartilage and the retroarticular process articulates with the articular process of the palatoquadrate dorsolaterally. The lateral part of Meckel's cartilage is less curved compared to earlier stages. The whole cartilage and its processes chondrify simultaneously at stage C2 (Fig. 2), where most cartilages of *D. scovazzi* have left the chondroblast state.

#### Palatoquadrate

**Fig. 4:**
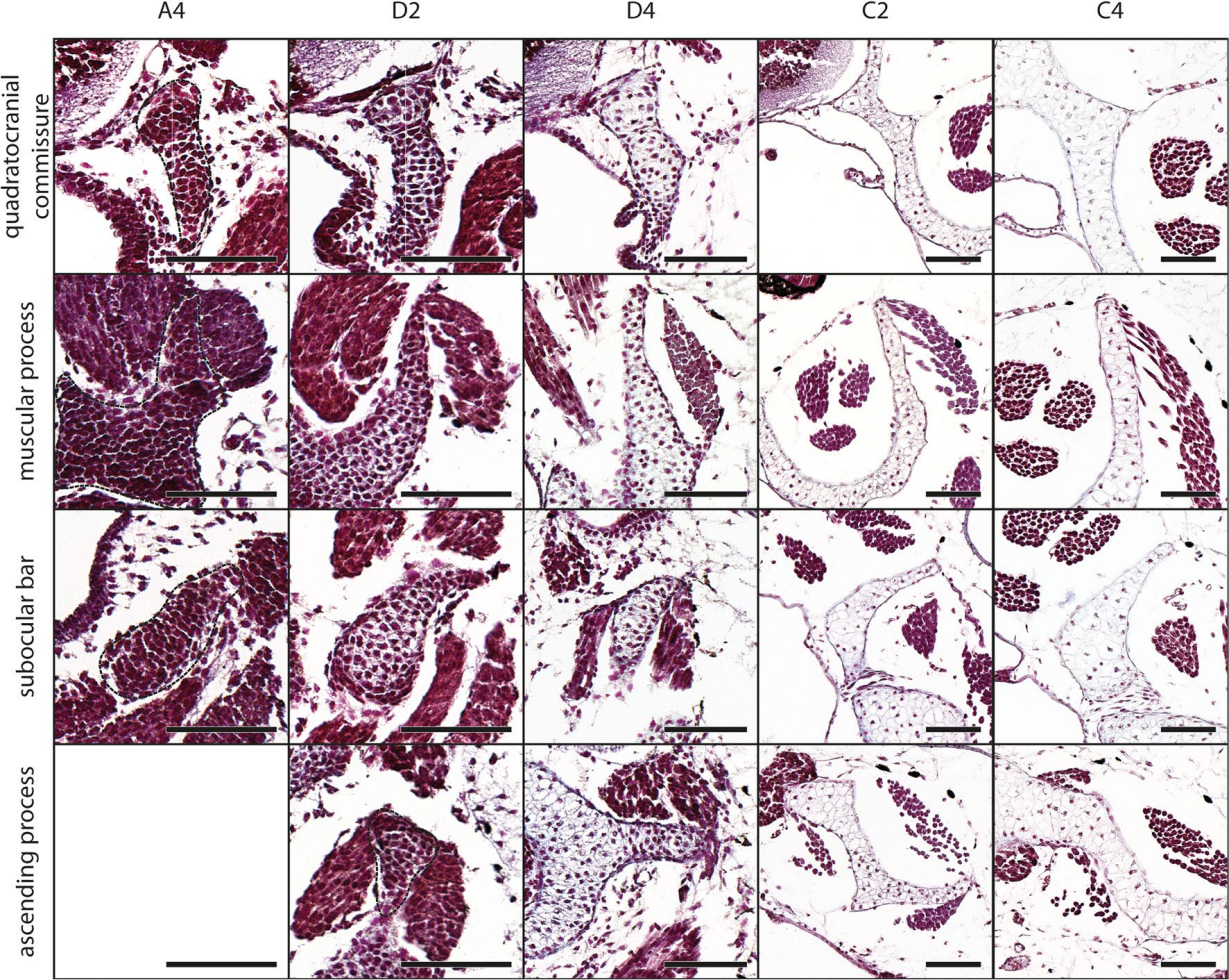
*D. scovazzi*, cartilaginous development of the palatoquadrate and its components. Transverse histological sections of Stage A4 (first column), Stage D2 (second column), Stage D4 (third column), Stage C2 (fourth column) and Stage C4 (fifth column) show the chondrification process of the quadratocranial commissure, the muscular process, the subocular bar and the ascending process. Difficult observable structures are highlighted by dashed lines. Scale bars indicate 100 μm

The palatoquadrate is the dorsal component of the mandibular arch. The jaw joint is formed by the articular process of the palatoquadrate and the retroarticular process of Meckel’s cartilage. The articular process is the most anterior part of the palatoquadrate. The posterior parts, like the muscular process and the subocular bar, are the origin of numerous muscles which enable jaw and eye movement. The palatoquadrate borders the neurocranial structures laterally and is connected to them via the quadratocranial commissure anteriorly and the ascending process posteriorly. In several larval amphibians there is a third neurocranium anchoring structure, the larval otic process, which is missing in *D. scovazzi*. The mesenchymal Anlage of each palatoquadrate is bipartite and arises at stage A2 (Fig. 3b). The Anlage of the quadratocranial commissure is flanking the pharyngeal cavity laterally whereas the Anlagen of the articular process and the hyoquadrate process border the Anlage of the mandibular levator muscles ventrally. Until stage A3, the Anlage of the muscular process extends dorsolaterally, while the Anlage of the subocular bar extends posteriorly. The lateral Anlage of the quadratocranial commissure is now connected to the ventral Anlage of the palatoquadrate. The second neurocranial anchoring process, the ascending process, arises at stage A5 during further posterior extension of the Anlage of the palatoquadrate. The first parts of the palatoquadrate to differentiate into chondroblasts at stage D3 are the mesenchymal Anlagen of both neurocranium anchoring structures (Fig. 4). The muscular process extends further dorsally and is now flanked by the developing orbitohyoideus muscle laterally and mandibular levator muscles medially. Three processes (muscular, articular, hyoquadrate) and the subocular bar differentiate into chondroblasts at stage D4 (Fig. 4). At the same stage the ligamentum tectum becomes visible as connective tissue between the dorsal tip of the muscular process and the neurocranium. While the Anlagen appear in a more or less antero-posterior pattern, the different parts of the palatoquadrate do not chondrify in anterior to posterior direction. Instead, the quadratocranial commissure and the subocular bar chondrify first at stage C1, whereas all other parts chondrify simultaneously at stage C2 (Fig. 4). The subocular bar becomes wider and flatter during further development. It extends posteriorly but never reaches the otic capsule.

#### Basihyal

The basihyal is a small, median cartilage anterior to the large ceratohyals. No muscles attach onto it. The mesenchymal Anlage develops at stage D4 between the anterior tips of the anterior processes of the ceratohyals (Fig. 3a). The Anlage remains small during differentiation. At stage C2 the chondroblasts differentiate. At stage C4 the whole Anlage consists of chondrocytes which form a small, rounded plate-like cartilage.

#### Ceratohyal

**Fig. 5:**
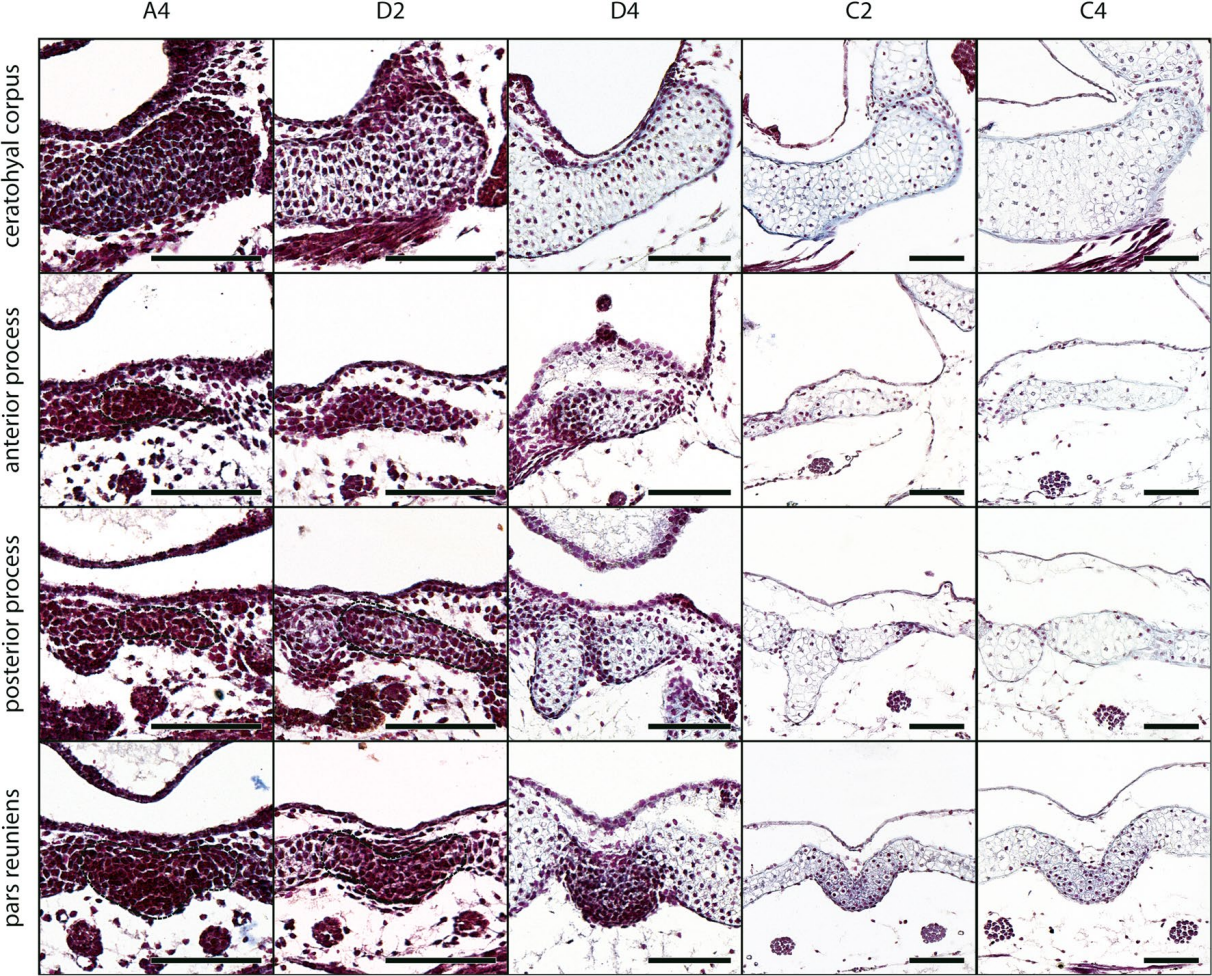
*D. scovazzi*, cartilaginous development of the ceratohyal and its components. Transverse histological sections of Stage A4 (first column), Stage D2 (second column), Stage D4 (third column), Stage C2 (fourth column) and Stage C4 (fifth column) show the chondrification process of the ceratohyal corpus, the anterior process, the posterior process and pars reunines. Difficult observable structures are highlighted by dashed lines. Scale bars indicate 100 μm

The ceratohyal of *D. scovazzi* is a paired cartilage which each consists of a corpus, an anterior, a lateral, and a posterior process as well as an articular condyle. An anterolateral or postcondylar process is not present. The two separate mesenchymal Anlagen of the cartilage fuse via the median pars reuniens. The corpus, the lateral process, and the articular condyle are present as mesenchymal Anlagen at stage A2 (Fig. 3a, b). The corpus arises ventrally to the pharyngeal cavity. The Anlage of the orbitohyoideus muscle extends laterally to the lateral process. The articular condyle is situated ventrally to the Anlage of the hyoquadrate process of the palatoquadrate. A gap between the two Anlagen closes at stage A3 when the mesenchymal Anlage of the pars reuniens, which connects both Anlagen anteriorly, arises. From stage A3 onwards only one Anlage of the ceratohyal is present. The mesenchymal Anlagen of the anterior and posterior processes also appear at this stage. The ceratohyal further widens laterally and the posterior process bulges posteriorly. At stage D1 the corpus is the first structure in which cells differentiate into chondroblasts. The articular condyle, and lateral and posterior processes are made of chondroblast by stage D4 (Fig. 5), followed by the anterior process at stage D5 and the pars reuniens at stage D6 (Fig. 3a). The lateral process becomes more pointed whereas the anterior process remains inconspicuous and flat. The pars reuniens extends posteriorly and is continuous with the basibranchial. All parts of the ceratohyal chondrify at stage C2 (Fig. 5), except the pars reuniens, which chondrifies at stage C3. The ceratohyal is horizontally oriented and the lateral parts bend posteriorly. The hyoquadrate process of the palatoquadrate and the articular condyle of the ceratohyal form a joint at stage C2.

#### Basibranchial

In *D. scovazzi* the basihyal is located medially between the ceratohyals. The pars reuniens connects the ceratohyal to the anterior part of the basihyal. The posteroventral part bears the inconspicuous urobranchial process. The mesenchymal Anlage of the basibranchial arises at stage A3 ventral to the Anlage of the midsagittal pars reuniens of the ceratohyal. The Anlage extends posteriorly towards the Anlage of the hypobranchial plate. At stage D1 the mesenchymal Anlage of the urobranchial process develops at the posteroventral margin of the basibranchial. At stage D4 the Anlage of the basibranchial consists of chondroblasts and the oblique subarcual muscle (M. subarcualis obliquus) attaches on the lateral surface of the mesenchymal urobranchial process. The latter differentiates into chondroblasts at stage D5. The precartilaginous Anlage is a small rod which further elongates posteriorly until stage C2 where the chondroblasts of the basibranchial and its urobranchial process differentiate into chondrocytes. The anterior part of the basibranchial is from now on fused to the ceratohyal.

#### Branchial basket

**Fig. 6:**
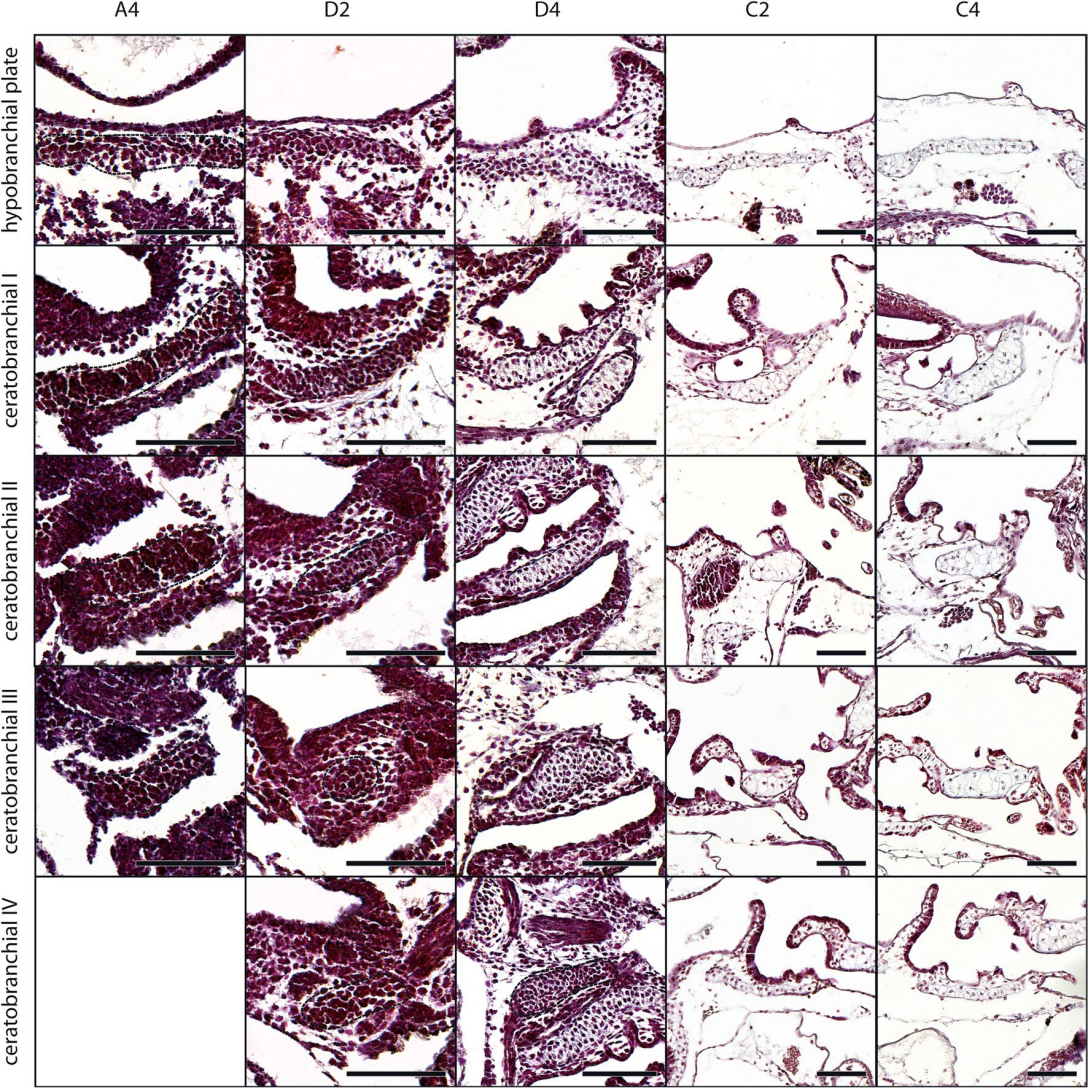
*D. scovazzi*, cartilaginous development of the branchial basket. Transverse histological sections of Stage A4 (first column), Stage D2 (second column), Stage D4 (third column), Stage C2 (fourth column) and Stage C4 (fifth column) show the chondrification process of the hypobranchial plate and the ceratobranchial I-IV. Difficult observable structures are highlighted by dashed lines. Scale bars indicate 100 μm

The branchial basket of *D. scovazzi* consists of four ceratobranchials on each side, which are fused ventromedially via the proximal commissures I-III and dorsolaterally via the terminal commissures I-III. The midsagittal hypobranchial plate is continuous with the medial ends of all ceratobranchials. Anteriorly the plate is a single element, but it gives rise to two posterior extensions which are only connected via connective tissue. A distinct hypobranchial element, spicules, or craniobranchial commissures are not present in the investigated stages of *D. scovazzi*. The mesenchymal Anlagen of the hypobranchial plate and ceratobranchial I arise at stage A2 (Fig. 6a). The bilateral Anlagen of the hypobranchial plate are beneath the ventromedial surface of the pharyngeal cavity. The Anlagen of the ceratobranchial I lie laterally to the Anlagen of the hypobranchial plate bordering the ventral and ventrolateral surface of the pharyngeal cavity. Parallel to the Anlage of ceratobranchial I, but more posterior, develops the mesenchymal Anlage of ceratobranchial II at stage A3. After that the Anlage of the proximal commissure I becomes visible at stage A4 between the medial ends of ceratobranchials I and II. At the same stage and posterior to the Anlage of ceratobranchial II the Anlage of ceratobranchial III develops as a small horizontally oriented rod. The ceratobranchials further extend laterally and medially and their medial ends get closer to the hypobranchial plates while their lateral ends slightly bend dorsally. At stage D2 and D3 all remaining parts of the branchial basket became visible as mesenchymal Anlagen (Fig. 3a). Between the stages D4 (Fig. 6) and D6 all structures differentiate into chondroblasts. The ceratobranchials and the hypobranchial plate are still relatively thin, but they become thicker during the following stages. The chondroblasts of the hypobranchial plate and ceratobranchial I are the first to differentiate into chondrocytes at stage C1 followed by the remaining structures of the branchial basket at stage C2 (Fig. 6). The general shape of the branchial basket is acquired at this stage and the whole basket grows bigger during further development.

#### Neurocranium

**Fig. 7:**
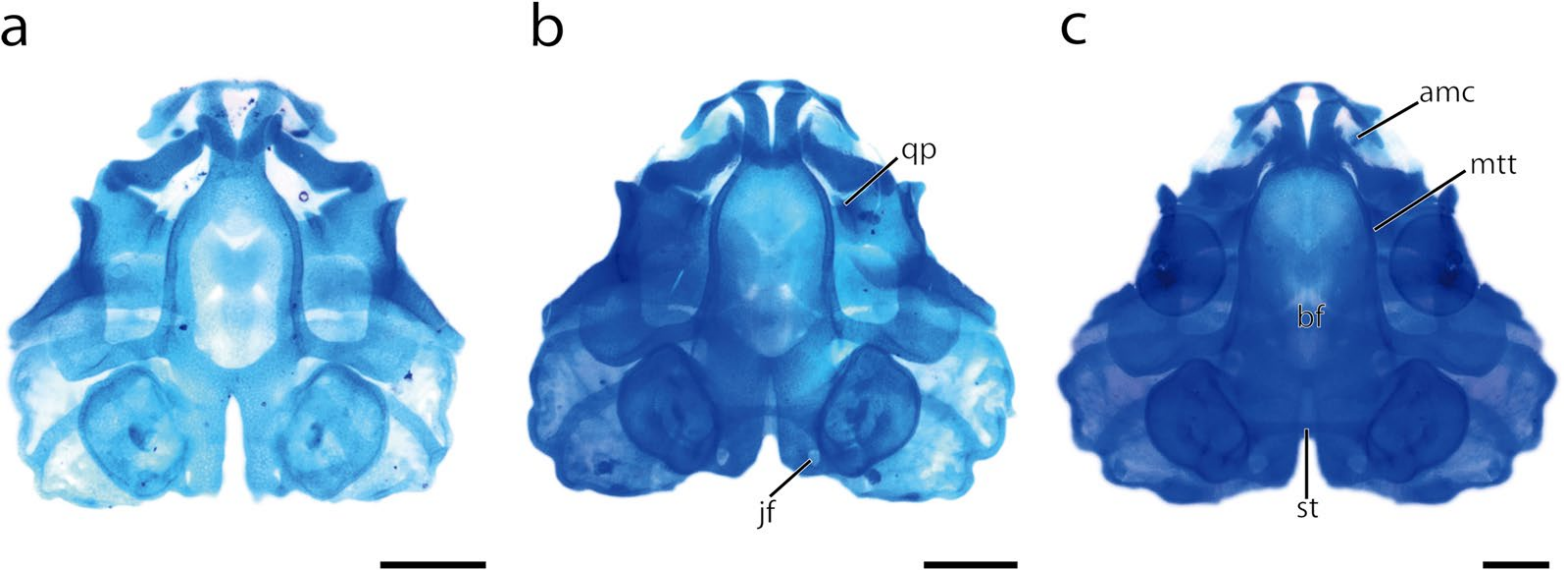
*D. scovazzi*, development of the cartilaginous head skeleton of larvae after all major cartilages are present. Note the development of the synotic tectum and the quadratoethmoid process as well as the closure of the basicranial fenestra. Shown are cleared and stained specimens of stage Go28 (**a**), Go32 (**b**) and Go34 (**c**) Scale bars indicate 0,5 mm. amc, admandibular cartilage; bf, basicranial floor; jf, jugular foramen; mtt, medial tectal taenia; qp, quadratoethmoid process; st, synotic tectum

The Neurocranium of *D. scovazzi* consists of a single ethmoid plate and bilaterally present a trabecular horn, an orbital cartilage, a marginal tectal taenia flanking the brain, a parachordal and an otic capsule which surrounds the inner ear. First traces of cartilaginous development are found within the neurocranium at stage A1. In that stage, the two posteriormost structures, the otic capsule and the parachordal, are the first structures which develop as mesenchymal Anlagen. The parachordals are visible as two small spheres lateral to the notochord and the lateral part of the otic capsule is visible posterolaterally to the Anlage of the parachordal. The two mesenchymal Anlagen of the trabecular horn develop anterodorsally to the pharyngeal cavity at stage A2. The parachordals extend laterally and anteriorly whereas the trabecular horn extends posteriorly, and the orbital cartilage emerges as mesenchymal Anlage from its posterior border at stage A3. At stage A5 the posterior extending orbital cartilage merges with the Anlage of the parachordal. The latter contains chondroblasts at stage D3. Orbital cartilage and trabecular horn differentiate into a precartilaginous Anlage at stage D4 (Fig. 3b). At this stage numerous Anlagen differentiate into chondroblast containing Anlagen. The mesenchymal Anlage of the otic capsule differentiates into chondroblasts at stage D6 and merges with the Anlage of the parachordal medially. Simultaneously the mesenchymal Anlage of the marginal tectal taenia occurs as dorsal outgrowth of the cranial trabecula. Stage C1 is marked by the differentiation of the chondroblasts of the trabecular horns, the orbital cartilages and the parachordal cartilages into chondrocytes. They are among the first cartilages which are fully chondrified. At stage C2 the posterior parts of the trabecular horns fuse medially and form a plate-like structure which borders the brain anteriorly, the ethmoid plate. At the same stage the chondroblasts of the otic capsules and the marginal tectal taenia differentiate into chondrocytes. Additionally, the mediodorsal surfaces of the otic capsules are connected via the mesenchymal Anlage of the synotic tectum, which differentiates into a chondroblast containing Anlage at stage C5. Until Gosner [26] stage 34 the synotic tectum chondrifies and the basicranial fenestra closes (Fig. 7).

### Comparison of *D. scovazzi*, *X. laevis*, *B. orientalis*

We used data from earlier studies describing the chondrogenesis in *X. laevis* [21] and *B. orientalis* [25] to compare our recent results. We scored additional cartilaginous features in both species to build a uniform database for future comparison with a wider range of species. We scored 11 additional cartilaginous features for *X. laevis* and 22 for *B. orientalis* (Tables 2, 3). Some structures, like the admandibular cartilage and urobranchial process of the basibranchial are not present in *X. laevis* and *B. orientalis* and are therefore not suitable for a comparison with *D. scovazzi*. In *X. laevis* and *B. orientalis* the external morphology does not give many indications about the developmental sequence of chondrification of the cranial skeleton. However, in *D. scovazzi* there is a clear correlation between the external morphology and the progress of skeletogenesis; i.e., in specific Gosner stages specific chondrocranial features are present. Another peculiarity in *D. scovazzi* is the simultaneous transition of numerous structures from mesenchymal to prechondrogenic Anlage at stage D4 (Fig. 3a,b) and from prechondrogenic to chondrogenic Anlagen at stage C2 (Figs. 2, 3–6). In *X. laevis* and *B. orientalis* the differentiation is more stepwise and extended over several stages.

**Table 2:**
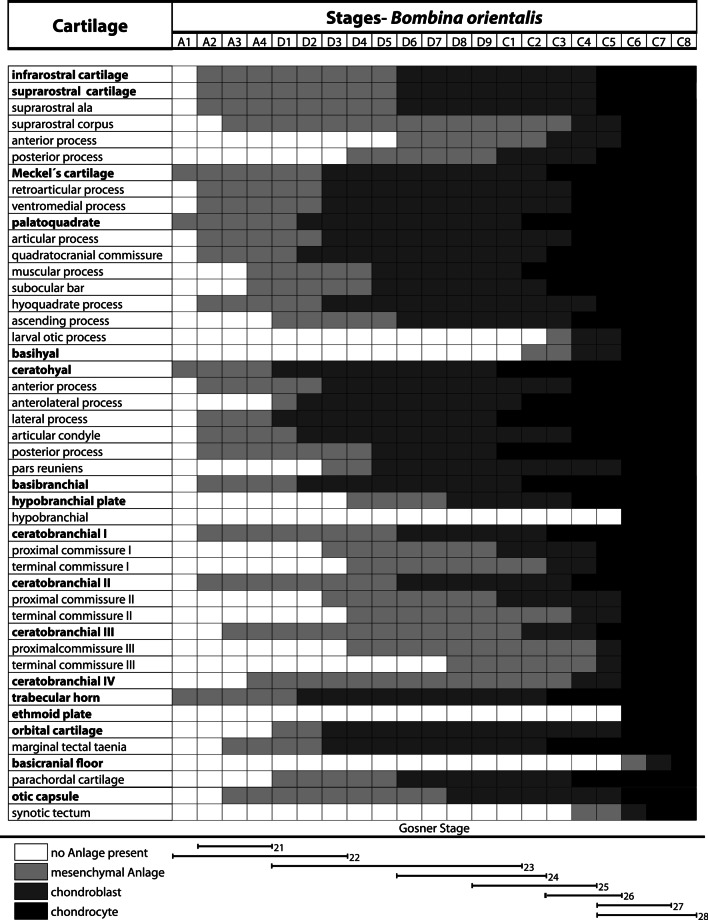
*X. laevis*, sequence of occurrence of cartilaginous development in tadpoles

**Table 3:**
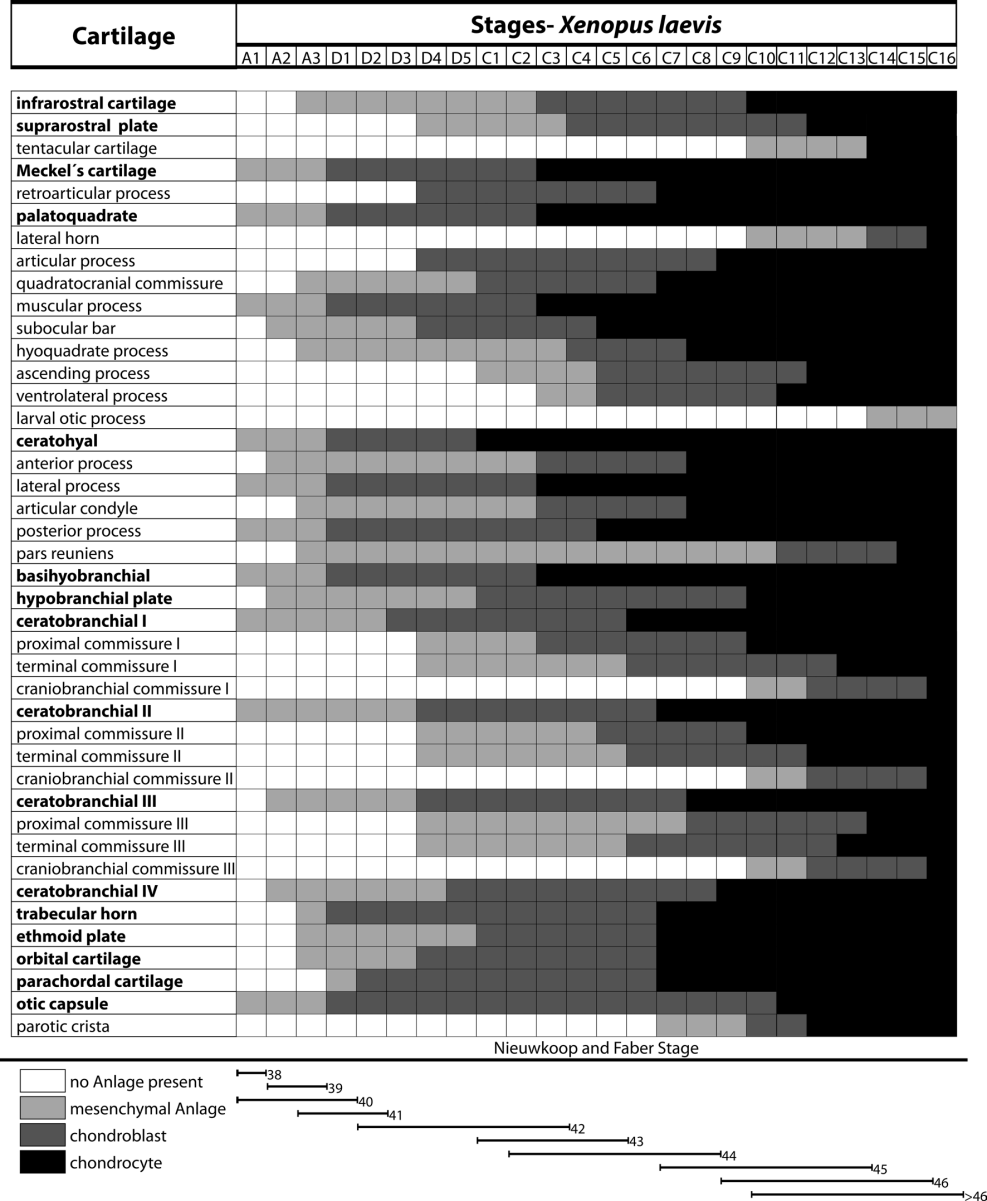
*B. orientalis*, sequence of occurrence of cartilaginous development in tadpoles

Not only is it possible to conclude from the developmental stage to the state of cranial skeletogenesis in *D. scovazzi*, but the species also shows some sequential features not observed in the other species studied here. The mesenchymal Anlage of the infrarostral cartilage is among the last appearing Anlagen during the cranial skeletogenesis in *D. scovazzi*, while it is one of the earliest mesenchymal Anlagen of the cranial skeleton in *X. laevis* and *B. orientalis*. Additionally, only one infrarostral Anlage is present in *D. scovazzi* instead of two as observed in the other two species. The chondrification of the various elements of the ceratohyal and the branchial basket happens simultaneously in *D. scovazzi* but successively in *X. laevis* and *B. orientalis*. Simultaneous chondrification can be a sign of limited resolution in one species as compared to the others. However, since all three species were studied in a comparable range of stages it could also be that the elements really chondrify simultaneously or at least in a faster sequence in *D. scovazzi*, than in the other investigated species.

While there are characters where *D. scovazzi* stands out, there are also several shared features of cartilaginous development with its close relative *B. orientalis*. Regarding the suprarostral cartilage, in both species the alae differentiate before the corpus of the suprarostral and the mesenchymal Anlage of the posterior process develops as the last element of the suprarostral cartilage. Additionally, the chondrification of the whole cartilage only takes two stages. Another commonality is that the ventromedial and retroarticular processes of Meckel's cartilage differentiate simultaneously. The basihyal and the synotic tectum develop late in both species. It is not possible to compare the suprarostral of *X. laevis* with the other species as the suprarostral plate consists of the suprarostral, the trabecular horns and the anterior process of the ethmoid plate, while there are no distinguishable ala.

There were also shared features of all three compared species *D. scovazzi*, *B. orientalis,* and *X. laevis;* one is that the development of the palatoquadrate is relatively similar. The palatoquadrate develops early and no anterior to posterior developmental sequence was observed. However, several other structures develop in an anterior to posterior sequence. Such a sequence is present during the development of the neurocranium-anchoring structures of the palatoquadrate (quadratocranial commissure, ascending process, larval otic process). The larval otic process, which is not present in *D. scovazzi*, is the last part of the palatoquadrate that chondrifies in *X. laevis* and *B. orientalis*. Furthermore, an anterior to posterior differentiation pattern is also observable in the branchial basket of all three species, including the ceratobranchials I-IV as well as the proximal and terminal commissure I-III.

### Additionally notes for *Xenopus* and *Bombina*

The sequences were updated from Lukas and Olsson [21, 25] and the updated tables are presented here (Tables 2, 3).

## Discussion

### Larval skeletal characters and their phylogenetic implications

Most of the skeletal features of *Discoglossus scovazzi* are congruent with descriptions made in *D. pictus* and *D. sardus* [27–29]. But some features are different in *D. scovazzi*. The hypobranchial plate is fused to ceratobranchial I in contrast to the synchondrotic articulation between hypobranchial I (the anterior part of the hypobranchial plate) and ceratobranchial I described in *D. pictus* [27]. The presence of the spicules I-III, a postcondylar process at the posterior margin of the ceratohyal and the presence of transversal tectal taenia were mentioned as defining features of the genus *Discoglossus* [28, 29]. However, these structures are absent in the investigated stages of *D. scovazzi* (here studied up to Gosner [26] stage 29 = Go29). If the absence is an apomorphic trait of *D. scovazzi* or if these structures appear later during development (Haas [29] studied Go37 and Púgener [28] Go34 tadpoles) requires further investigation.

An admandibular cartilage is present in a similar condition as described in other species of *Discoglossus* [28, 30], the closely related *Alytes* [29, 30], and the basalmost neobatrachian *Heleophryne* [31–33]. Among Discoglossoids, species of *Bombina* lack this cartilage [25, 29]. Therefore, the presence of the admandibular cartilage might be an apomorphic feature of the Alytidae (comprising Alytinae and Discoglossinae) and distinguish them from the Bombinatoridae, as also suggested by recent molecular based phylogenies [34–36]. Further features which separate both clades are the absence of the larval otic process of the palatoquadrate and the lack of a synchondrosis between the trabecular horns and the suprarostral cartilage in Alytids [28, 30, 37] whereas both are present in Bombinatorids [25]. An inconspicuous additional feature of Alytids is the consistent width of the trabecular horns [30] while the width of them is terminally expanded in Bombinatorids [25, 37].

A common feature of all Discoglossoids investigated so far is the presence of two posterior processes of the ala of the suprarostral cartilage [21, 25, 29, 30]. This feature qualifies as apomorphic trait for the Discoglossoidea. Infrarostrals which are not fused are present in Alytinae, Discoglossinae, Bombinatoridae, and the basal Ascaphidae [25, 30, 38]. Therefore, this unfused condition may be synapomorphic for all anurans. The suprarostral alae and the suprarostral corpus are separated in *Ascaphus truei* [39], whereas both are connected in the majority of the so-called archaeobatrachians. Therefore, even if a separated suprarostral appears frequently among neobatrachians, this may reflect the ancestral anuran state of this cartilaginous structure [37]. The presence of a hyoquadrate process in all basal anurans also qualifies as a synapomorphic trait [37].

*X. laevis* has a specialized filter feeding larvae and while Pipidae are Mesobatrachia, several of the above-mentioned features cannot be found. Paired infrarostrals are present in ascaphids, discoglossoids, scaphiopodids, pelodytids and pelobatids [25, 30, 37, 38, 40, 41]. *Megophrys* and *Xenopus* have fused infrarostrals [21, 42], whereas *Hymenochirus, Pseudhymenochirus,* and *Rhinophrynus* have no infrarostrals at all or they are fused to Meckel´s cartilage [21, 43, 44]. *X. laevis* also differs from the cartilaginous development of *B. orientalis* and *D. scovazzi* because of the bipartite origin of the palatoquadrate. *X. laevis* possesses two mesenchymal Anlagen of the palatoquadrate. The anterior Anlage extends posteriorly during further development and gives rise to the muscular process, the quadratocranial commissure and the subocular bar. The posterior Anlage grows anteriorly and gives rise to the ventrolateral process and the ascending process [21]. If we consider the tentacular cartilage of *X. laevis* as elongated lateral outgrowth of the fused suprarostral alae, then *X. laevis* contradicts the Discoglossoid sequence where the suprarostral corpus develops after the suprarostral alae. Instead, the suprarostral corpus, which is part of the suprarostral plate, chondrifies before the suprarostral alae (tentacular cartilage) in *X. laevis* [21].

### Developmental sequence

The viscerocranium of chondrichthyans [17, 18], teleosts [14], and sturgeons [20] develops in strict anterior to posterior direction. We have shown that basal anurans differ from this ancient pattern [21, 25]. In *B. orientalis* and *X. laevis* the ceratohyal, an element of the hyoid arch, is the first cartilage to chondrify during development while the infrarostral and suprarostral cartilages, elements of the mandibular arch, develop much later. We assumed that the early development is linked to the importance of the ceratohyal as the central element of the buccal pump which draws water into the buccal cavity and enables proper inspiration and expiration. Furthermore, we assumed that the early development of the ceratohyal may be an apomorphic trait for the Lalagobatrachia (all anurans except the basal Ascaphidae and Leiopelmatidae). Both assumptions are challenged by the sequence observed in *D. scovazzi*. The ceratohyal has an interesting developmental trajectory. The mesenchymal Anlage of the ceratohyal develops before the mesenchymal Anlage of the suprarostral ala and subocular bar, but simultaneously with the mesenchymal Anlage of the quadratocranial commissure, ceratobranchial I and hypobranchial plate. Chondroblasts appear earlier in the ceratohyal than in those five elements, but all those elements finish their chondrification before the ceratohyal. Furthermore, studies comparing muscle developmental sequences in vertebrates suggest that the mandibular arch-derived structures regularly appear simultaneously or after hyoid arch-derived structures [12], which would support the hypothesis proposed by Miyashita [13] that the mandibular arch only became secondarily integrated with the ancestral pharyngeal arches (hyoid and branchial arches) in the transition that lead to the last common ancestor of crown-group Gnathostomata and only became secondarily similar to them. With the current available developmental sequences it is not possible to verify the ancestral sequence in amphibians but further studies are underway.

There is also no strict anterior to posterior chondrification pattern within the viscerocranium of *D. scovazzi*. Hyoid arch elements (e.g., basihyal) chondrify after elements of the branchial arch (e.g., ceratobranchial I-IV) and mandibular arch elements (e.g., infrarostral cartilage) chondrify after elements of the branchial arch I (e.g., ceratobranchial I). Therefore, we can confirm that the development of the viscerocranium of anurans is a complex mosaic and does not follow a strict directional pattern. The ceratobranchials I-IV develop more simultaneously in *D. scovazzi* than in *B. orientalis*, *X. laevis*, *Rana temporaria*, *Bufo cinereus,* and *Hyla sp*. [21, 25, 45, 46] where they develop in a stepwise anterior to posterior sequence. However, the chondrification of the neurocranium anchoring processes of the palatoquadrate (quadratocranial commissure and ascending process) follows a clear anteroposterior sequence in *D. scovazzi* as was described before in several other anuran species [21, 25, 45, 46].

In *Ascaphus truei* the lateral parts of the suprarostral chondrify later than the medial parts [39]. In *B.orientalis* and in *D. scovazzi* the lateral alae develop before the medial corpus of the suprarostral cartilage [25]. The suprarostral is not present as a separate element in *X. laevis* as it is fused with trabecular horns, anterior process of ethmoid plate; the medial suprarostral plate develops before the lateral tentacular cartilages. This leads to four possible scenarios. (1) The developmental sequence of the suprarostral observed in *A. truei* is an apomorphic trait of all anurans and the sequence observed in *D. scovazzi* and *B. orientalis* is a derived trait of the Lalagobatrachia. (2) The developmental sequence of the suprarostral observed in *A. truei* is an apomorphic trait of all anurans and the sequence observed in *D. scovazzi* and *B. orientalis* is a derived trait of the Discoglossoidea. (3) The developmental sequence of the suprarostral observed in *A. truei* is a derived trait of the Ascaphidae and the sequence observed in *D. scovazzi* and *B. orientalis* is the apomorphic trait of all anurans. (4) The developmental sequences are apomorphic for Ascaphidae and Discoglossidae and the ancestral sequence is different. Adding further sequential investigations on cartilaginous development of anurans, salamander and caecilians should shed light on this question.

The mandibular arch derived cartilages, Meckel’s and palatoquadrate, originate from a single mesenchymal anlage, which is the ancestral gnathostome condition [17, 47]. The mesenchymal anlage separates into the ventral anlage of the Meckel’s cartilage and the lateral anlage of the palatoquadrate. The later chondrification of both cartilages is independent of each other. In anurans, two separate distinct mesenchymal Anlagen of Meckel's cartilage and palatoquadrate exist at the earliest investigated specimens [21, 25, 45]. However, since several developmental studies have shown that the continuous anlage of the first branchial arch will give rise to the upper and lower jaw elements as a response to genetic patterning [e.g., Dlx pattern, 48], it is reasonable to assume that this is the case in amphibians too.

Comparing the general appearance of branchial arch derived Anlagen and ignoring the individual processes of specific cartilages (Tables 1–3) then we can make following observations: In *D. scovazzi* the mandibular, hyoid, and first branchial arch Anlagen develop first followed by stepwise addition of the branchial arches II, III, and IV. In *B. orientalis* the mandibular and hyoid anlagen appear first, followed by anlagen of ceratobranchial I and II, and a stepwise addition of the other two ceratobranchials. In *X. laevis* mandibular, hyoid, and first and second branchial arch Anlagen develop first followed by third and fourth ceratobranchial Anlagen in the next step. This anteroposterior development of pharyngeal arches with simultaneous occurrence of mandibular and hyoid arch derivatives was also described for several amphibians, including *B. orientalis* and *X. laevis* [12, 49], but in fishes the mandibular arch anlagen often appear after the hyoid arch Anlagen (*Scyliorhinus canicula:* [12]; *Polypterus senegalus:* [50]; *Danio rerio:* [51]; *Neoceratodus forsteri* [11]*:*). In aves and in Theria (marsupials and placental mammals) there seems to be some variability (*Gallus domesticus*: [9]; *Coturnix coturnix*: [52]; *Monodelphis domestica*: [53]; *Mus musculus*: [52]). The evolutionary significance of this appearing pattern is not obvious to us and might require the analyses of a wider range of taxa to evaluate for eventual functional or developmental constraints.

The neurocranium of several osteichthyans (bony fishes; e.g., Japanese medaka: [14], sturgeons: [20], coelacanth: [23], Australian lungfish: [55]), “reptiles” [16, 19, 22, 23, 56, 57] and birds [24] develop in posterior to anterior direction. Otic capsules, parachordals, and cranial trabeculae are the first centers of chondrification and extend anteriorly to build the neurocranial elements in these species. Once again, anurans differ from this gnathostome pattern. Parachordals (C1) and otic capsules (C2) are among the first structures to chondrify in *A. truei* and *D. scovazzi* ([39], present work). These posterior parts of the neurocranium extend anteriorly, while the anterior parts of the neurocranium, the trabecular horns and the orbital cartilage extend posteriorly until both parts meet and fuse. Neurocranial structures develop later in *X. laevis* and *B. orientalis*, but the anterior growth of posterior elements and the posterior growth of anterior elements is also present [21, 25]. The late development of the synotic tectum is not only a feature of anurans [21, 25, 39, 45] but also present in avians and the Australian lungfish [24, 55]. Additionally, the chondrification of the otic capsule from lateral to medial seems to be conserved among anurans [21, 25, 39, 45].

## Conclusions

We compared the timing of the cranial chondrification of three basal anuran species and hypothesize the following features of anuran cartilage formation:

1. The viscerocranial elements do not chondrify in the presumably ancestral anterior to posterior direction.
2. The neurocranial elements do not develop in the presumable ancestral posterior to anterior direction. Instead, the posterior elements extend anteriorly and the anterior elements extend posteriorly direction until both parts meet.
3. The neurocranium-anchoring processes of the palatoquadrate chondrify in anterior to posterior direction; first the quadratocranial commissure, then the ascending process, and last the larval otic process.
4. The infrarostral cartilage and the proximal commissure I chondrify simultaneously.
5. The palatoquadrate and basibranchial chondrify simultaneously.
6. Anterior to posterior developmental sequences can be observed best in the branchial apparatus development.

The here presented sequences in anurans support that the posterior to anterior direction of development and growth of the parachordal as well as the initial development of the lateral wall of the otic capsule are shared gnathostome features.

## Methods

### Husbandry and staging

Fertilized eggs of *Discoglossus scovazzi* were provided by a private breeder. Approximately 20 eggs per petri dish were cultured in 0.1X modified Barth's saline [58]. They were kept at temperatures between 18 and 25 °C to ensure that every stage of interest can be collected (3–5 tadpoles every 2 h). All specimens (embryos and larvae) were staged according to the simplified staging table for anuran embryos and larvae [26] and denominated as “Go stages.” A developmental series was established by collecting embryos and larvae from defined stages between Go 20 and Go 34 (n = 156). Anesthesia was performed using 1% tricaine methanesulfonate (MS-222) according to the animal welfare protocols at the Friedrich-Schiller-University Jena. Specimens were fixed in 4% phosphate-buffered formalin (PFA). Histological sections and cleared-and-stained larvae are kept at the Institute of Zoology and Evolutionary Research, Friedrich-Schiller-University, Jena, Germany.

### Tissue staining

The PFA-fixed specimens were dehydrated, embedded in paraffin, and serial sectioned at 7 μm thickness using a rotary microtome (Microm, HM 355 S). The sections were stained according to Heidenhain's Azan technique [59]. Images were taken with an Hitachi HV-F202SCL camera mounted on an Zeiss AxioScan Z1 microscope operated with Zen 3.1 software. The clearing-and-staining procedure followed the protocol by Dingerkus and Uhler [60] with the exception that no alizarin red was used due to the absence of bones. Cleared-and-stained specimens were examined with a Zeiss Stemi 11 and images were taken by an attached camera (ColorView) operated by AnalySIS software.

### Image processing

The digitized images (TIFF-format) of the respective histological sections were exported to Fiji Software [61]. The section images of each individual were stacked and aligned using first the least squares (rigid) and second the elastic non-linear block correspondence mode from the TrakEM2 plugin for Fiji [62]. Elastically aligned stacks were exported as tagged image files (TIFF). Segmentation of the different skeletal structures was performed in Amira 6.0.1. 3D analysis software (FEI Visualization Sciences Group). Polygonal surfaces were rendered and then exported to Wavefront OBJ file format for further processing in Autodesk Maya 2021 (Autodesk, Inc.). Surfaces were smoothed, polygonal counts reduced, and the surfaces arranged. For the final composition, coloring and rendering of images, Autodesk Mudbox 2021 (Autodesk, Inc.) was used. All images were edited and arranged using Adobe Photoshop CS6 and Adobe Illustrator CS6 (Adobe Inc.).

### Scoring

We defined and scored a total of 75 cartilaginous structures (cartilages, processes, etc., incl. joints) in each investigated specimen of *D. scovazzi* (44 out of 75 are present)*.* To enable a proper comparison, earlier descriptions from *X. laevis* and *B. orientalis* [21, 25] were updated to enable maximal comparable structures. Terminology used here follows the guidelines introduced by Haas [29] for cartilaginous features (anglicized terms). The following different states of cartilaginous development were scored: (1) cartilaginous structures are absent; (2) mesenchymal Anlagen are visible as condensed cell clusters; (3) chondroblasts form condensed precartilaginous cell clusters with clearly visible nuclei. (4) chondrocytes rich in cytoplasm and bordered by a clearly visible perichondrium are present. All structures were scored after the first appearance principle. If a certain state was visible within the respective structure, the whole structure was scored in this state even if parts of the structure remained in an earlier state. Additionally, we defined different stages of development. Each stage is defined by a specific combination of cartilages in different cartilaginous developmental states. A1 (Anlage 1) starts with the appearance of the first mesenchymal Anlage, D1 (Differentiation 1) starts with the appearance of the first Anlage which contains chondroblasts and stage C1 (Cartilage 1) starts with the appearance of the first chondrocyte containing cartilage. The following enumeration of stages is the result of additional cartilaginous structures entering different states.

## Data Availability

Sufficient data generated and analyzed during this study are included in this published article [and its supplementary information files]. The complete datasets used and/or analyzed during the current study are available from the corresponding author on reasonable request.
